# Current Trend of Marine Carbohydrate-Containing Compounds with Medicinal Properties

**DOI:** 10.3390/md19060331

**Published:** 2021-06-08

**Authors:** Irina M. Yermak, Viktoria N. Davydova

**Affiliations:** G.B. Elyakov Pacific Institute of Bioorganic Chemistry, Far Eastern Branch, Russian Academy of Sciences, 100 Let Vladivostoku Prosp., 159, 690022 Vladivostok, Russia

Carbohydrates are most abundant biomolecules on Earth and, also, the most complex biomolecules in terms of structure. Marine carbohydrate-containing substances obtained from marine sources such as algae, microbes, and animals are usually biodegradable and biocompatible and exhibit biological properties that contribute to the discovery of a wide range of new bioactive substances with special pharmacological properties of interest to medicine. Carbohydrate-based compounds include glycans, glycoproteins, proteoglycans, glycolipids, and low-molecular and complex glycosides of differential origins. Marine glycans are remarkable molecules, playing a determinant role in biological processes. All cells, including human cells, have carbohydrates on their surfaces, known as the glycocalyx. The glycocalyx is a useful target for personalized medicine, including finding new biomarkers for diseases such as cancer and for patient stratification in clinical trials. The functional studies of carbohydrates concern molecular recognition such as carbohydrate–lectin or glycoside–enzyme interactions, cell recognition (normal and pathological), viral adhesion/penetration, and many other phenomena. Carbohydrate recognition plays a vital role in the activation and function of the immune system. The production and applications of marine carbohydrates as therapeutic agents are increasingly important topics of intensive research. A modern study of carbohydrates includes the development of glycotechnologies in the field of diagnosis and therapy of diseases and nutrients. The interest in the study of marine polysaccharides with therapeutic purposes relies on the possibility of developing novel approaches of less invasive and more personalized treatments. Many of these polysaccharides allow loading lower drug dosages, which may lead to a drastic reduction of the side effects caused by the drugs. The use of marine polysaccharides in the systems of drug delivery in tissue engineering and regenerative medicine is rapidly developing due to the numerous functional groups in their molecules, strong water absorption, favorable physicochemical properties, safety, low cost, wide distribution in nature, and biological activity. In addition, the structure of polysaccharides can be relatively easily modified in order to synthesize derivatives with desirable characteristics for drug delivery. Carbohydrate complexes are well-tolerated macroorganism systems and can be used in various fields, such as drug delivery systems; matrices for cell cultivation and enzyme immobilization; and materials for the reconstruction of bone, cartilage, cardiac, and dental tissues.

The Special Issue “Marine Carbohydrate-Based Compounds with Medicinal Properties” of the open access journal *Marine Drugs* (ISSN 1660-3397) ran from February 2020 to January 2021. In total, it consisted of 12 articles devoted to various aspects of marine carbohydrate-containing substances and the recent experimental studies carried out on their basis. In this Special Issue, we discussed the biological aspects and structural features of the various carbohydrate-based compounds of marine origin endowed with potential biomedical and biotechnological applications. The isolation of marine biologically active carbohydrate compounds and relevant studies on their structures and properties are important for the adding knowledge about molecular diversity in nature and the creation of medicines and other useful products.

Marine polysaccharides and their derivatives as soluble substances have promising prospects for medical use. Many microorganisms, including marine bacteria, secrete extracellular polysaccharides called exopolysaccharides (EPSs). EPSs have a lot of promise in cell therapy and tissue engineering. In the Special Issue, two articles were devoted to the study of EPSs isolated from marine bacteria. Since their natural tropism in targeting malignant tumors and their metastasis, EPSs have been recently considered as a vector to be combined with theranostic radionuclide pairs [1]. Antimetastatic properties in both murine and human osteosarcoma cell lines (POS-1 and KHOS) were evidenced by using exopolysaccharide derivatives, produced by the *Alteromonas infernus* bacterium [2]. Muñoz-Garcia et al. developed a strategy for further use as a therapeutic vector of EPS derivatives. First, the authors had to make sure that EPS might keep the ability of targeting cancer cells even when complexed with scandium (Sc). For this reason, in vitro studies concerning both the cell proliferation and cell viability of various tumor cell lines were realized. The cell index of human osteosarcoma (MNNG/HOS), human melanoma (A375), human lung cancer (A549), human glioblastoma (U251), and human breast cancer (MDA231) were monitored over a week using XCELLigence^®^ technology. The tested complexes exhibited an antiproliferative effect; this effect was more effective compared to EPS alone. This increase of the antiproliferative properties was explained by a change in conformation of the EPS complexes due to their polyelectrolyte nature that was induced by complexation. The results, obtained by the authors, are very promising and reveal that EPS can be coupled to scandium for improving its biological effects and, also, suggest that no major structural modification occurs on the ligand [2].

The work of Gargouch et al. [3] demonstrated the biological activity of different molar mass exopolysaccharides (EPS) from *Porphyridium marinum* and its oligomers prepared by a high-pressure homogenizer. The analyses of the polymer compositions and viscosity measurements were conducted on all samples in order to propose hypotheses involving the activities caused by the intrinsic properties of the polymers. All EPS samples were tested for different biological activities, i.e., antibacterial, antifungal, and antibiofilm activities on *Candida albicans*, as well as for their effects on the viability of murine breast cancer cells. The results of these researchers showed the ability of EPS to suppress the proliferation of Gram (+) and Gram (−) bacteria, as well as the formation of a biofilm of *Candida albicans* in low concentrations. However, low molar masses were found to be more effective for antiproliferative activity against breast cancer cells. Depending on the biological activity tested, this study also disclosed that these biological activities could be due to their molar masses and their viscosity, as well as their composition (probably their content in sulfate and uronic acids). Thus, this study provided strong arguments to consider EPS from *P. marinum* as a natural source of antibacterial, antibiofilm, and anticancer products that are useful in pharmaceutical formulations and food industries as a natural preservative. Nevertheless, this study constituted a first step to evaluate the potential of EPS from *P. marinum* in a drug development approach.

Despite the numerous polysaccharide structures elucidated, the diversity of EPS seems largely underestimated, and the tools to predict their structures are still absent. The article by Drouillard et al. [4] concerned the structural elucidation of the polysaccharide secreted by *Vibrio alginolyticus* CNCM I-5035. *Vibrio alginolyticus* secretes an exopolysaccharide used as ingredient in the cosmetic industry under the trademark Epidermist. It is appreciated for its ability to improve the physical and chemical barrier functions of the skin by notably increasing the keratinocyte differentiation and epidermal renewal. Analyses of the native and alkali-treated polysaccharides, as well as the detailed characterization of purified di- and trisaccharides, demonstrated that the polysaccharide was composed of a repetition unit of three residues: d-galactose (d-Gal), d-*N*-acetylglucosamine (GlcNAc), and l-*N*-acetylguluronic acid, of which 30% (*m/m*) was acetylated in position 3. The complete structure of the polysaccharide was resolved giving the repetition unit: (→3)-α-d-Gal-(1→4)-α-l-GulNAcA/α-l-3OAc-GulNAcA-(1→4)-β-d-GlcNAc-(1→). This repetition unit was very similar to that found in the lipopolysaccharide extracted from *Pseudoalteromonas nigrifaciens* KMM161 [5]. The structure of the polysaccharide investigated by Drouillard et al. was the third exopolysaccharide structure secreted by a *V. alginolyticus* strain [6]. The composition of the repetition unit determined, as well as the sequence of residues, had no similarities, suggesting that the biosynthetic pathways of these polysaccharides probably have no common ancestor. The structural diversity found in the *V. alginolyticus* strains was also true for all the strains of the *Vibrio* genus. The Carbohydrate Structure Database [7] has entries describing polysaccharides—including secreted polysaccharides or lipopolysaccharides—of the genus *Vibrio*. The structural diversity of the polysaccharides suggests a very high plasticity of the polysaccharide biosynthesis pathway of the strains belonging to the genus *Vibrio.*

Due to of their substantial diversity and rich composition of biologically active compounds, algae are of considerable interest to the pharmaceutical industries and medicine. The structures and biological activities of polysaccharides from brown, red, and green algae were discussed in several papers of the Special Issue. The structure and immunostimulatory effect of two sulfated and pyruvate galactans from green alga *Caulerpa* *cupressoides* var. *flabellata* were studied by Barboza et al. [8]. According to NMR spectroscopy, both galactans were composed primarily of 3)-β-d-Galp-(1→3) units. Pyruvate groups were also found, forming five-membered cyclic ketals as 4,6-*O*-(1′carboxy)-ethylidene-β-d-Galp residues. Some galactose residues were sulfated at C-2. One of these sulfated polysaccharides (SP) had some galactose units sulfated at C-4. An analysis of the effects of these SP on the macrophages resulted in the production of nitrogen oxide (NO), reactive oxygen species(ROS) and proinflamatory cytokines, which indicated that sulfation at the C-4 position is not essential for the immunomodulatory activity of these galactans. The immunostimulating activity of *C. cupressoides* galactans showed their practical potential in the development of new biomedical products.

A protective effect on colitis in mice induced by dextran sulfate sodium (DSS) of the sulfate *Ulva* polysaccharide with low molecular weight (LMW-ulvan), prepared using the enzymatic method, was studied by Li et al. [9]. MW-ulvan had a molecular weight of 2.56 kDa and contained (1→3,4)-linked Rha, (1→4)-linked Xyl, and (1→4)-linked GlcA, with small amounts of (1→4)-linked Rha residues; the sulfate substitution was at the C-3 of Rha. MW-ulvan reduced the inflammatory infiltration and damage, which can be attributed to the enhancement of the antioxidant defense system in mice. It was also suggested that the improvement of LMW-ulvan in DSS-induced colitis might be closely related to its protective effect on the intestinal mucosal barrier by increasing the expression of tight junction proteins. Thus, LMW-ulvan could further inhibit the abnormal immune response in the intestinal mucosa, thus ameliorating the mucosal barrier function and intestinal mucosal permeability. These results indicated that LMW-ulvan may be a promising application for inflammatory bowel disease.

The work of Vigors et al. [10] was devoted to the study of the effect of an algal polysaccharide extract that was used in the diet of pigs on the productivity of animals—in particular, the immune and microbial profile of their gastrointestinal tract. The authors used algae extracts to improve the performance and digestive health in postweaning pigs. The weaning process is a stressful event in the animal’s life, characterized by intestinal and immune dysfunctions that result in impaired feed intake, health, and growth. Previously, this group of authors showed that feeding extracts such as laminarin (LAM) and fucoidan in the immediate postweaning period inhibit multiple beneficial physiological adaptations in the gastrointestinal tract that can improve pig performance during this difficult time [11]. In this regard, the authors continued their work and studied the effects of feeding with seaweed extracts enriched with laminarin (LAM) and fucoidan (FUC) on the performance indicators of the animals, gut microbiological profile, and transcriptome profiles up to 35 days after weaning. While neither extract had beneficial effects on the animal performance, the LAM supplementation had a positive influence on the intestinal health through alterations in the gastrointestinal microbiome and increased butyrate production. 

The brown algae also synthesized alginates that are widely used in the food and pharmaceutical industries. Alginate oligosaccharide (AOS) is an oligomer of alginate, which has the characteristics of low relative molecular weight, good solubility, high safety, and high stability. The alginate lyase has unique advantages in the preparation of alginate oligosaccharides and processing of brown algae. Alginate lysis is adopted to catalytically degrade alginate into AOS by β-elimination under relatively mild and controllable conditions. The gene Alg2951, encoding a PL7 family alginate lyase with exo/endo-type activity, was cloned from a novel marine bacterium *Alteromonas portus* HB161718T and then expressed in *Escherichia coli* by Huang et al. [12]. The recombinant Alg2951 in the culture supernatant reached an activity of 63.6 U/mL, with a molecular weight of approximately 60 kDa. The results showed that Alg2951 was a cold-adapted PL7 alginate lyase with a significant preference toward polyG. Moreover, Alg2951 was shown to be a NaCl- and KCl-activated enzyme with exolytic and endolytic activity. Meanwhile, it specifically degraded polyG and sodium alginate but had almost no activity on polyM. The authors suggested that Alg2951 could catalyze the hydrolysis of sodium alginate to produce monosaccharides and trisaccharides. The enzymatic hydrolysates displayed good antioxidant activity by assays of the scavenging abilities towards radicals (hydroxyl and ABTS+) and the reducing power. Due to its cold-adapted and dual exo/endo-type properties, Alg2951 can be a potential enzymatic tool for industrial production.

The ability to synthesise acid polysaccharides as carrageenans (CRGs) and agar is a most interesting property of red seaweeds. Intensive studies have shown that CRGs can be regarded not only as foodstuff ingredients but, also, as drugs because of a wide spectrum of biological and physiological activities [13]. The inhibitory effects of carrageenans (CRGs) on lipopolysaccharides (LPS) induced inflammation in a mouse model of endotoxemia and, in the complex therapy of patients with enteric infections of *Salmonella* etiology, were appreciated by Yermak et al. [14]. CRGs were able to increase the synthesis of anti-inflammatory interleukin 10 (IL-10) in vitro, and, at low concentrations, their activity in the mixture with LPS was higher. The protective effect of different structural types of CRGs against *Escherichia coli* LPS was studied in vivo by monitoring the biochemical and pathomorphological parameters that respond most adequately to any acting stressor, including bacterial endotoxin. Less-sulfated κ- and κ/β-CRGs and the food supplement “Carrageenan-FE” increased the nonspecific resistance of mice to *E. coli* LPS at the expense of the inhibition of the processes of thymus involution, adrenals hypertrophy, thyroid atrophy, hypercorticoidism, glycogenolysis, and lactate acidosis. The authors suggested that the nonspecific resistance of the organism to *E. coli* LPS induced by CRG may be due to both the immunomodulatory effect of CRGs and their influence on the macromolecular structure of LPS. Additionally, in the current study, the estimation of the therapeutic action of “Carrageenan-FE” in a complex therapy of patients with enteric infections of *Salmonella* etiology was evaluated. “Carrageenan-FE” restored the system of hemostasis and corrected some biochemical indicators and parameters in the immune systems of the patients. These results allow to hope for a practical application of CRGs for lowering the endotoxemia level in patients under the development of the infectious process caused by Gram-negative bacteria.

One of the well-known polysaccharides is chitosan. In this Special Issue, the medico-biological properties of chitosan and its derivatives were discussed in three articles. The mechanism of healing of burn wounds under the action of a chitosan (CS) hydrogel containing gentamicin (GT) was systematically explored by Yan et al. [15]. The wound dressing based on the chitosan–gentamicin conjugate had antibacterial properties and good cytocompatibility and hemocompatibility. The wound healing experiment showed a synergic effect between CS and GT and the highest recovery rates in the late phase of trauma healing. The animals in the CS–GT group had the thickest dermis, dense dermal mesenchyme, and spindle-shaped fibroblasts, indicating optimal tissue repair. A further analysis showed that the CS–GT hydrogel promoted the synthesis of total proteins in the granulation tissue of the skin, led to the facilitation of collagen fibrogenesis, decreased the expression of inflammatory cytokines, and, ultimately, accelerated wound healing.

Lan et al. [16] evaluated the effects of the dietary supplementation of chitosan oligosaccharides (COS) on the intestinal integrity, oxidative status, and the inflammation response with a hydrogen peroxide (H_2_O_2_) challenge. A dietary COS supplementation decreased the duodenum and ileum mucosal IL-6 level and jejunum mucosal tumour necrosis factor-α (TNF-α) level, inhibited the expression of IL-6 in the jejunum and ileum and TNF-α in the jejunum and ileum, and increased the duodenum and ileum mucosal IL-10 levels. The results of this research group suggested that COS can maintain the integrity of the intestine during oxidative stress by modulating the oxidative status of the bowel and release of inflammatory cytokines. These results suggested that COS could maintain the intestinal integrity under oxidative stress by modulating the intestinal oxidative status and release of inflammatory cytokines. Dietary COS supplementation may be an effective nutritional strategy to alleviate the detrimental effects of oxidative stress.

Complexes on the basis of carbohydrates are often prepared to improve their functional properties. This approach allows easily adapting complex characteristics for specific medical applications. The immunotropic activity of polyelectrolyte complexes (PEC) of chitosan with polysaccharides of red seaweed–κ-carrageenan (κ-CRG) of various compositions was assessed in comparison with the initial polysaccharides in comparable doses [17]. The highest activity of CS and κ-CRG, as well as their soluble PEC, was observed in a histamine-induced exudative inflammation directly related to the activation of phagocytic cells, i.e., macrophages and neutrophils. The ability of PEC to scavenge NO depends on the content of the κ-CRG in the PEC. The ability of the PEC to induce the synthesis of proinflammatory (TNF-α) and anti-inflammatory (IL-10) cytokines in peripheral blood mononuclear cells was determined by the activity of the initial κ-CRG, regardless of their composition.

Glyceroglycolipids are widely distributed in plants, microalgae, and cyanobacteria and present bioactivities and pharmacological activities and can be widely used in the pharmaceutical industry. A study by Xu and Miao [18] provided further insights into the glyceroglycolipid metabolism, as well as the scientific basis for glyceroglycolipid synthesis optimization and cyanobacteria glyceroglycolipids utilization via metabolic engineering. The authors showed that there are 12 differentially expressed transcriptional regulators that could be potential candidates related to glyceroglycolipid regulation, according to a transcriptome analysis. The transcriptome analysis also suggested post-transcriptional or post-translational regulations in glyceroglycolipid synthesis.

Thus, this Special Issue covered a series of experimental studies recently carried out in the field of marine carbohydrate-containing compounds. In their publications, scientists from Ireland, Russia, France, China, the Republic of Korea, and Brazil discussed new results obtained from the isolation and study of marine polysaccharides and their derivatives and complexes. The wide spectrum of biological activities of these compounds, such as immunomodulatory, antibacterial, anticancer, anti-inflammatory, antibiofilm, and antioxidant, obtained in experiments in vitro and in vivo showed the prospects for their practical use in medicine (their practical potential in the development of new biomedical products).

As guest editors, we are thankful to all the scientists from the diverse research institutes and universities who contributed to the success of the Special Issue “Marine Carbohydrate-Based Compounds with Medicinal Properties”.
